# Human intracranial pulsatility during the cardiac cycle: a computational modelling framework

**DOI:** 10.1186/s12987-022-00376-2

**Published:** 2022-11-01

**Authors:** Marius Causemann, Vegard Vinje, Marie E. Rognes

**Affiliations:** 1 Department of Numerical Analysis and Scientific Computing, Simula Research Laboratory, Kristian Augusts gate 23, 0164 Oslo, Norway; 2 Department of Mathematics, University of Bergen, P. O. Box 7803, 5020 Bergen, Norway

**Keywords:** Intracranial pulsatility, Cerebral blood flow, Intracranial pressure, Cerebrospinal fluid, Interstitial fluid, Finite element model, Poroelasticity

## Abstract

**Background:**

Today’s availability of medical imaging and computational resources set the scene for high-fidelity computational modelling of brain biomechanics. The brain and its environment feature a dynamic and complex interplay between the tissue, blood, cerebrospinal fluid (CSF) and interstitial fluid (ISF). Here, we design a computational platform for modelling and simulation of intracranial dynamics, and assess the models’ validity in terms of clinically relevant indicators of brain pulsatility. Focusing on the dynamic interaction between tissue motion and ISF/CSF flow, we treat the pulsatile cerebral blood flow as a prescribed input of the model.

**Methods:**

We develop finite element models of cardiac-induced fully coupled pulsatile CSF flow and tissue motion in the human brain environment. The three-dimensional model geometry is derived from magnetic resonance images (MRI) and features a high level of detail including the brain tissue, the ventricular system, and the cranial subarachnoid space (SAS). We model the brain parenchyma at the organ-scale as an elastic medium permeated by an extracellular fluid network and describe flow of CSF in the SAS and ventricles as viscous fluid movement. Representing vascular expansion during the cardiac cycle, a prescribed pulsatile net blood flow distributed over the brain parenchyma acts as the driver of motion. Additionally, we investigate the effect of model variations on a set of clinically relevant quantities of interest.

**Results:**

Our model predicts a complex interplay between the CSF-filled spaces and poroelastic parenchyma in terms of ICP, CSF flow, and parenchymal displacements. Variations in the ICP are dominated by their temporal amplitude, but with small spatial variations in both the CSF-filled spaces and the parenchyma. Induced by ICP differences, we find substantial ventricular and cranial-spinal CSF flow, some flow in the cranial SAS, and small pulsatile ISF velocities in the brain parenchyma. Moreover, the model predicts a funnel-shaped deformation of parenchymal tissue in dorsal direction at the beginning of the cardiac cycle.

**Conclusions:**

Our model accurately depicts the complex interplay of ICP, CSF flow and brain tissue movement and is well-aligned with clinical observations. It offers a qualitative and quantitative platform for detailed investigation of coupled intracranial dynamics and interplay, both under physiological and pathophysiological conditions.

**Supplementary Information:**

The online version contains supplementary material available at 10.1186/s12987-022-00376-2.

## Introduction

The pulsating brain environment features a unique and dynamic interplay between blood influx and efflux, cerebrospinal fluid (CSF) flow in and between the cranial and spinal compartment, intracranial pressures (ICPs), brain tissue movement and interstitial fluid (ISF) flow. Alterations in the dynamics of ICP or CSF flow are associated with central nervous system disorders [62] such as hydrocephalus [34, 46], Alzheimer’s disease and multiple sclerosis [49]. Moreover, better understanding of CSF flow characteristics could play an important role for targeted drug delivery [43]. Progress in magnetic resonance imaging (MRI) has allowed for non-invasive measurements of CSF flow, blood flow, and brain tissue deformation [5, 50]. Over the last decade, computational modelling of brain mechanics have emerged as a promising complementary tool to obtain high fidelity and high resolution models and predictions of intracranial dynamics [37].

Computational studies of intracranial pulsatility have mainly focused on either the brain parenchyma [26, 27, 60] or the flow of CSF through the ventricular system and the spinal and cerebral subarachnoid spaces (SAS) [30, 35, 55, 61]. These models have successfully been applied to improve understanding of CSF related disorders and to explain pulsatile pumping in perivascular spaces [32]. However, such *decoupled* approaches do not fully account for the close interactions between the brain tissue and the surrounding CSF, and the potential exchange between CSF and ISF. In contrast, *coupled* fluid-structure interaction models allow for simultaneous computation of flow and pressure in the CSF-spaces as well as the solid displacement and stresses in the brain parenchyma. Linninger et al [36] proposed a model of CSF flow in the SAS and ventricles coupled with porous media flow through the brain parenchyma driven by an oscillatory inflow boundary condition at the choroid plexus. Sweetman et al [53] introduced a 3D model of CSF flow with fluid-structure interaction driven by a moving lateral ventricle wall. Tully and Ventikos [56] investigated the coupling of poroelasticity and free fluid flow in the cerebral aqueduct using an idealized brain model. Gholampour [24] used a coupled model of CSF flow and brain viscoelasticity – again driven by a CSF source in the lateral ventricles to compare flow patterns in healthy and hydrocephalic subjects. To the authors’ knowledge, only a few studies have investigated intracranial dynamics with poroelastic and fluid flow models involving both the parenchyma and cranial SAS (see e.g. [37] for a comprehensive review), and all prescribed pulsatile motion have had its origin at the ventricles or choroid plexus. However, if brain-wide expansion of blood vessels is the main driver of pulsatility [5, 62], a local source or boundary condition may be inadequate to properly induce pulsatile intracranial motion observed in vivo [62].

Based on multi-modal MR imaging, Balédent [5] proposed that cardiac cycle-induced intracranial pulsatility is driven by the following sequence of events. During systole, arterial blood flow into the brain exceeds the venous outflow, the brain expands, ICP increases, and CSF is displaced into the spinal canal. Subsequently, during diastole, venous outflow dominates the vascular dynamics, leading to a decrease of ICP and a reversal of CSF flow. A key question is whether and to what extent computational models can integrate this view of intracranial dynamics, driven by the cardiac-induced expansion of blood vessels in the brain tissue [5], with clinical observations of ICP [20], ICP differences [21, 61], and CSF flow.

In this paper, we therefore propose a computational model of intracranial dynamics coupling the pulsatile motion of CSF, brain tissue and ISF during the cardiac cycle. We represent the brain parenchyma at the organ-scale as an elastic medium permeated by an extracellular network saturated by CSF/ISF. Flow of CSF in the SAS and ventricles is modelled as a viscous fluid under low Reynolds numbers i.e. via the Stokes equations. Pulsatile cerebral blood flow was not explicitly computed in the model, but distributed over the brain parenchyma acting as a brain-wide source term to initiate motion of brain tissue and ISF. The scope of the present work is thus limited to the dynamical interaction between brain tissue, ISF and CSF. Specifically, the model predicts the brain displacement, intracranial pressures within the parenchyma, in the SAS, and in the ventricular system, and CSF and ISF flows. Several model variations (e.g. parameter regimes) were also tested to assess the sensitivity to different parameters. Overall, our computational results agree well with clinical observations of ICP, stroke volumes, and brain displacements, and thus introduces a promising computational approach to study intracranial pulsatility in response to intraparenchymal blood flow.

## Methods

### Domains and boundaries

**Fig. 1:**
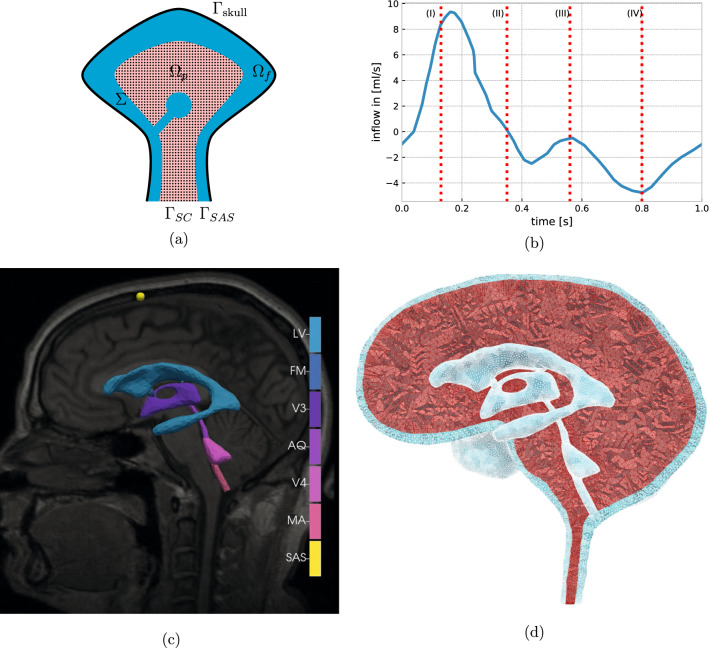
**a** Sketch of the domains representing the brain parenchyma ($\Omega _p$, pink) and the CSF-filled spaces ($\Omega _f$, blue). The interface of both domains is denoted by $\Sigma$. Additionally, the boundaries $\Gamma _{\mathrm{skull}}$ at the skull, $\Gamma _{\text {SC}}$ at the spinal cord and $\Gamma _{\text {SAS}}$ at the spinal SAS are highlighted; **b** Net blood inflow during the cardiac cycle with four different phases: (I) early systole - high net blood inflow; (II) end of net blood inflow phase; (III) brain equilibrium phase (arterial inflow and venous outflow almost match); (IV) high net outflow of blood (data extracted from Balédent [5]); **c** The MRI image used for the mesh generation and the segmented parts of the ventricular system: *LV* lateral ventricles, *FM* foramina of Monro, *V3* third ventricle, *AQ* aqueduct of Sylvius, *V4* fourth ventricle, *MA* median aperture, *SAS* probe point in the subarachnoid space; **d** sagittal view of the mesh, displaying the ventricular system, cranial SAS (both light blue) brain parenchyma (red)

We represent the brain parenchyma as a three-dimensional domain $\Omega _p$, and the surrounding CSF-filled spaces by $\Omega _f$ (Fig. 1a). These two domains share a common boundary $\Sigma = \Omega _f \cap \Omega _p$ with normal vector ${{\mathbf {n}}}$, pointing from $\Omega _f$ to $\Omega _p$ on $\Sigma$ and outwards on the boundary $\partial \Omega$. Further, $\Gamma _{\mathrm{skull}}$ denotes the outer boundary of the CSF space where the rigid skull encloses the cranial cavity (Fig. 1a). The lower boundary of the domain (at the C3 level) is split into two segments: the caudal continuation of the spinal cord is labeled $\Gamma _{SC}$, while $\Gamma _{SAS}$ describes the boundary to the spinal SAS. Hence, our model is spatially limited to the intracranial cavity and the uppermost part of the spine, and we do not compute CSF flow in the entire spinal canal. To obtain a computational mesh of both the parenchyma and the cranial CSF-filled spaces, we manually segmented the *full head MRI scan* data set (1x1x1.3 mm resolution) provided by Slicer3D [23, 31], and extracted the constituents of the ventricular system, the cranial SAS including the main subarachnoid cisterns, and the brain parenchyma (Fig. 1d). Only some small features such as the lateral apertures, the tentorium cerebelli or smaller cisterns could not be resolved due to image resolution and computational limits. The surfaces of the segmented regions were meshed using the Surface Volume Meshing Toolkit (SVMTK) [59]. The volumes and diameters of the relevant mesh substructures, as listed in Table 1, are within clinically reported ranges. The computational mesh consists of 4526016 mesh cells, 796303 mesh vertices and a maximal (minimal) cell diameter of $6.7\,$mm ($0.2\,$mm).

**Table 1:** Comparison of the generated computational head model and experimentally determined values in healthy subjects with respect to the dimensions of the brain’s substructures; C3 is the third cervical vetrebra level of the spine

Substructure dimension	This study	Literature value	References
Lateral ventricles volume	$24.01\,$ml	$9.82\,$ml (normal) 250.2 ml (hydrocephalic)	Linninger et al [36]
Third ventricle volume	$3.60\,$ml	$2.48\,$ml	Linninger et al [36]
Fourth ventricle volume	$2.69\,$ml	$3.31\,$ml	Linninger et al [36]
Subarachnoid space volume	$292.08\,$ml	$179\,$ml (only cranial)	Chazen et al [15]
Aqueduct diameter	2.88 mm	1.5 to 3.0 mm	Haines and Mihailoff [28]
Spinal canal (C3) diameter	12 mm	9.4 to 17.2 mm	Ulbrich et al [57]
Spinal coord (C3) diameter	7 mm	6.0 to 9.6 mm	Ulbrich et al [57]
Brain parenchyma total volume	$1369.54\,$ml	1130 ml (women) 1260 ml (men)	Cosgrove et al [17]

### Governing equations

The brain parenchyma We regard brain tissue as a linear poroelastic medium permeated by a single fluid network representing an extracellular CSF/ISF-space. The equations of linear poroelasticity express conservation of momentum for the solid elastic matrix and the mass conservation of a diffusive flow within the medium [10]. Due to its robustness in case of materials close to the incompressible limit or with low storage capacity, we chose a three-field formulation, based on the displacement ${{\mathbf {d}}}$, fluid (pore) pressure $p_p$ and the additional total pressure $\phi$, which is defined as $\phi = \alpha p_p - \lambda \text { div } {{\mathbf {d}}}$ [33, 41]. With the infinitesimal strain tensor ${\mathbf {\epsilon (d)}} = \frac{1}{2}(\nabla {{\mathbf {d}}} + \nabla {{\mathbf {d}}}^T)$ and a volume source term *g*, the equations read as follows:

1a $$ - {\mathbf {div}} [2 \mu _s \epsilon ({{\mathbf {d}}}) - \phi {{\mathbf {I}}}] = 0 \;  \text { in } \Omega _p \times (0, T), $$

1b $$\phi - \alpha p_p + \lambda \text{ div } {{\mathbf {d}}}= 0 \; \text { in } \Omega _p \times (0, T), $$

1c $$\left( c + \frac{\alpha ^2}{\lambda } \right) \partial _t p_p - \frac{\alpha }{\lambda }\partial _t \phi -\text { div } \left( \frac{\kappa }{\mu _f} \nabla p_p \right) = g \; \text { in } \Omega _p \times (0, T). $$

Here, $\kappa$ represents the permeability, *c* the specific storage coefficient, and $\alpha$ the Biot-Willis coefficient. The identity operator is ${{\mathbf {I}}}$. The linear isotropic solid matrix is parameterized with the Lamé constants $\mu _s$ and $\lambda$, while the fluid permeating the pores has viscosity $\mu _f$.

CSF compartments We model the flow of CSF in the ventricular system and SAS by the time-dependent Stokes equations for the CSF velocity ${{\mathbf {u}}}$ and fluid pressure $p_f$. The Stokes equations represent flow under low Reynolds numbers typically observed in the CSF compartments; Howden et al [30] report an average Reynolds number of $Re_{av}=0.39$ with a maximum value of $Re_{max}=15$ in the CSF-filled spaces of the cranium during the cardiac cycle. Under these assumptions, the equations read as follows:

2a $$ \rho _f \partial _t {{\mathbf {u}}} - {\mathbf { div }}[2 \mu _f {\mathbf {\epsilon (u)}} - p_f {{\mathbf {I}}}] = 0 \; \text { in } \Omega _f \times (0, T), $$

2b $$ \text { div } {{\mathbf {u}}} = 0 \; \text { in } \Omega _f \times (0, T),$$

with the strain rate tensor ${\mathbf {\epsilon (u)}} =\frac{1}{2} \left( \nabla {{\mathbf {u}}} + \nabla {\mathbf {u^T}} \right)$, constant CSF density $\rho _f$, and constant CSF viscosity $\mu _f$.

### Net blood flow as a driver of pulsatility

We induce motion in the system via a vascular expansion through net flow of blood into the brain parenchyma, modelled by a prescribed pulsatile source term *g* in (1c). We define net blood flow as the difference between arterial blood inflow and venous blood outflow over time. As Biot’s equations include only one fluid network, we treat the net blood flow as a source term in this single fluid compartment. This simplification can be justified by the similarity of the effect of an inflow of blood and/or ISF: both lead to a volumetric expansion of the brain parenchyma and an increase of fluid pressure. With this approach the blood network is not explicitly computed, and we thus neglect material parameters such as blood viscosity and density.

We let the source term *g* vary in time, but be spatially uniform, and employ a net blood inflow time series measured in supine position from Balédent [5] (Fig. 1b). Net blood inflow results from subtracting venous outflow from arterial inflow at each corresponding time point over the cardiac cycle. The arterial inflow is the sum over the left and right internal carotid artery flow as well as the vertebral artery flow. The venous flow was computed as the sum over left and right internal jugular and epidural veins. To account for unmeasured small veins, the venous flow curve was adjusted to equalize total inflow and outflow over one cardiac cycle (see Balédent [5] for further details). The rapid inflow of arterial blood during early systole (phase I) increases the cranial blood volume, until venous outflow balances the arterial inflow, ending the net inflow of blood (phase II). Next, after a brief equilibrium (phase III), the venous outflow exceeds the arterial inflow (phase IV) and sets the cerebral blood circulation up for the next cycle. As both arterial and venous blood flow are prescribed, the model computes the response of CSF, ISF, and brain tissue for a given cerebral blood flow, and as such implements a one-way coupling between these compartments.

### Transmission, boundary and initial conditions

In contrast to the one-way coupling of the system with blood flow, the model fully accounts for the dynamic interplay of CSF, ISF and brain tissue motion. This two-way coupling is achieved via transmission (interface) conditions, which are imposed in addition to boundary and initial conditions.

Transmission Conditions

Based on first principles, we require the following equations to hold on the interface $\Sigma$ between the porous parenchyma and the CSF-filled spaces:

3a $$\begin{aligned} {{\mathbf {u}}}\cdot {{\mathbf {n}}}&= \left( \partial _t {{\mathbf {d}}} - \frac{\kappa }{\mu _f} \nabla p_p \right) \cdot {{\mathbf {n}}}&\text { on } \Sigma \times (0, T), \end{aligned}$$

3b $$\begin{aligned} \left( 2 \mu _f \epsilon ({{\mathbf {u}}}) - p_f {{\mathbf {I}}} \right) {{\mathbf {n}}}&= \left( 2 \mu _S \epsilon ({{\mathbf {d}}}) - \phi {{\mathbf {I}}} \right) {{\mathbf {n}}}&\text { on } \Sigma \times (0, T), \end{aligned}$$

3c $$\begin{aligned} -{{\mathbf {n}}} \cdot \left( 2 \mu _f \epsilon ({{\mathbf {u}}}) - p_f {{\mathbf {I}}} \right) {{\mathbf {n}}}&= p_p&\text { on } \Sigma \times (0, T), \end{aligned}$$

3d $$\begin{aligned} -{{\mathbf {n}}} \cdot \left( 2 \mu _f \epsilon ({{\mathbf {u}}}) - p_f {{\mathbf {I}}} \right) {\mathbf {\tau }}_i&= \frac{\gamma \mu _f}{\sqrt{\kappa }} \left( {{\mathbf {u}}} - \partial _t {{\mathbf {d}}} \right) \cdot {\mathbf {\tau }}_i&\text { on } \Sigma \times (0, T)&, \quad i=1,2. \end{aligned}$$

Here, to complement the normal ${{\mathbf {n}}}$, ${\mathbf {\tau }}_i$ ($i=1,2$) we define orthogonal tangent vectors to the interface, and $\gamma > 0$ is the slip rate coefficient, which is a dimensionless constant depending only on the structure of the porous medium. Here, (3a) enforces continuity of the normal flux on the interface, (3b) conserves momentum, while (3c) accounts for the balance of total normal stress. The last interface condition (3d) is the Beavers-Joseph-Saffman (BJS) condition, which states that the jump in the tangential velocities across the interface is proportional to the shear stress on the free flow side of the interface [8, 39, 48].

Boundary Conditions

Assuming a rigid skull, we set no-slip conditions on the skull boundary $\Gamma _{\mathrm{skull}}$:

$$\begin{aligned} {{\mathbf {u}}} = 0 \qquad \text { on } \Gamma _{\mathrm{skull}} \times (0, T) . \end{aligned}$$

For the spinal cord boundary $\Gamma _{\text {SC}}$, we assume no displacement and no flux:

$$\begin{aligned} {{\mathbf {d}}} = 0 \quad \text { and } \quad \frac{\kappa }{\mu _f} \nabla p_p \cdot {{\mathbf {n}}} = 0 \qquad \text { on } \Gamma _{\text {SC}} \times (0, T) . \end{aligned}$$

To represent the compliance of the spinal compartment, we assume an exponential relationship between ICP and additional volume [38, 54, 63]:

$$\begin{aligned} \left( 2 \mu _f \epsilon ({{\mathbf {u}}}) - p_f {{\mathbf {I}}} \right) \cdot {{\mathbf {n}}} = - {{\mathbf {n}}} \, p_0 \cdot 10^{ \Delta V_{\mathrm{out}}(t) / \mathrm{PVI_{SC}}} \qquad \text { on } \Gamma _{\text {SAS}} \times (0, T) . \end{aligned}$$

The pressure-volume index (${\mathrm{PVI}}_{\mathrm{SC}}$) represents a clinical measure of the compliance of the spinal compartment, $p_0$ is the initial pressure of the system and $\Delta V_{\mathrm{out}}(t)$ is the total additional volume of CSF in the spinal compartment. The latter equals the volume of CSF that has left the domain over the corresponding part of the boundary $\Gamma _{\mathrm{SAS}}$, and is calculated as follows:

$$\begin{aligned} \Delta V_{\mathrm{out}}(t) = \int _0^t \int _{\Gamma _{\text {SAS}}} {{\mathbf {u}}} \cdot {{\mathbf {n}}} \, \, {\mathrm {d}} s\, {\mathrm {d}} t. \end{aligned}$$

This allows for the pulsatile motion of CSF in and out of the domain.

Initial Conditions Finally, we assume that the system is initially at rest with an initial pore pressure $p_{0}$:

$$\begin{aligned} {{\mathbf {u}}}&= {{\mathbf {0}}} \qquad&\text { on } \Omega _f \times \{ 0 \}, \\ {{\mathbf {d}}}&= {{\mathbf {0}}}, \quad p_p = p_{0} \qquad&\text { on } \Omega _p \times \{ 0 \} . \end{aligned}$$

### Material parameters

Material parameters were selected based on literature values and are summarized in Table 2.

**Table 2:** Summary of material parameters, including references to values from previous studies. The Young’s modulus *E* and Poisson ratio $\nu$ are related to the elastic Lamé parameters as $\lambda =\frac{\nu E}{(1 -2 \nu )(1+\nu )}$ and $\mu _s = \frac{E}{2(1+\nu )}$

Parameter	Symbol	Value(s)	Unit	Reference	Value used in this study
Young modulus	*E*	$1895 \pm 592$ (wm) $1389 \pm 289$ (gm) 5000	Pa	Budday et al [12] - Smith and Humphrey [51]	1500
Poisson Ratio	$\nu$	0.479	-	Smith and Humphrey [51]	0.479
Density (brain tissue)	$\rho _s$	1081	$\text {kg}/\text {m}^3$	Barber et al [7]	1081
Density (CSF)	$\rho _f$	1007	$\text {kg}/\text {m}^3$	Barber et al [7]	1007
Biot-Willis coefficient	$\alpha$	1.0	-	Smith and Humphrey [51]	1.0
Permeability	$\kappa$	$10^{-17} - 4 \cdot 10^{-15}$	$\text {m}^2$	Holter et al [29]	$10^{-16}$
Storage coefficient	*c*	$4.47 \cdot 10^{-7}$ $3 \cdot 10^{-4} - 1.5 \cdot 10^{-5}$	$\text {Pa}^{-1}$	Chou et al [16] Guo et al [27]	$10^{-6}$
CSF/ISF viscosity	$\mu _f$	$0.7\cdot 10^{-3} - 10^{-3}$	$\text {Pa} \cdot \text {s}$	Bloomfield et al [11]	$0.8 \cdot 10^{-3}$
Spinal pressure-volume index	$\mathrm PVI_{SC}$	$2.94\pm 1.05$ $3.9\pm 2.5$	ml	Tain et al [54] Wåhlin et al [63]	3
Initial ICP	$p_0$	5 -15	mmHg	Rangel-Castillo et al [45]	4.5
Slip-rate coefficient	$\gamma$	$0.01 - 5$	-	Ehrhardt [19]	1

### Quantities of interest

Primary clinical quantities of interest are the ICP and CSF flow rates and volumes in the foramen magnum or across the aqueduct [62]. In our computational model, we identify the ICP as the (fluid) pressure $p_f$ in the CSF compartment(s) and as the total pressure $\phi$ in the parenchyma, which incorporates both the pore pressure and the stress exercised by the elastic matrix. We place virtual/computational pressure probe points inside the lateral ventricles, in the cranial SAS at the upper convexity of the skull, and inside the fourth ventricle (Fig. 1c). Flow rates within the ventricular system and into the spinal compartment are obtained by spatial integration of the computed CSF flow across boundaries between the different parts of the ventricular system or across the spinal external boundary, respectively. Specifically, we define the following set of quantities of interest:

i) the peak volumetric flow rate in the aqueduct,
ii) the aqueduct stroke volume, corresponding to the net volume of fluid pulsating back and forth in the aqueduct over the cardiac cycle (maximum of the cumulative flow volume),
iii) the peak tissue displacement,
iv) the (peak) transmantle pressure gradient, computed as the (peak) pressure difference between the virtual probe points in the cranial SAS and the lateral ventricles and divided by the distance between these points,
v) the temporal nadir-to-peak (i.e, diastolic to systolic) amplitude of pressure in the lateral ventricles,
vi) the spinal stroke volume, corresponding to the net volume of fluid pulsating back and forth into the spinal compartment over the cardiac cycle.

Results are reported from the last of three cardiac cycles to limit the influence of the initial data.

### Model variations

The effect of the model’s parameterization is of particular interest due to the uncertainty of the chosen parameters. Additionally, variations of material parameters offer insights into the relation of changing material characteristics (possibly caused by diseases or ageing) and alterations in the pulsatile motion of the brain. Since an extensive exploration of the parameter space of the model is out of scope for this work, we restrict our analysis to a collection of selected parameter deviations from the standard model (Table 3). For model A, we increase the pressure-volume index $PVI=10$ ml, which corresponds to a larger spinal compliance. Model B represents stiffer brain parenchyma (Young Modulus $E=3000$ Pa) while in model C we increase the compressibility of the brain (Poisson ratio $\nu =0.4$). Finally, model D features a greater storage coefficient ($c=10^{-5}\,\text {Pa}^{-1}$), which reduces the rise of pressure with additional fluid volume inside the poroelastic parenchyma and hence models larger intracranial compliance.

**Table 3:** Overview of the selected models, their deviation from the standard parameterization and the corresponding interpretation

Model	modified parameter	value	interpretation
Standard	-	-	
A	pressure-volume index	$PVI=10\,$ml	greater spinal compliance
B	Young Modulus	$E=3000\,$Pa	stiffer brain parenchyma
C	Poisson ratio	$\nu =0.4$	greater compressibility of brain tissue
D	storage coefficient	$c=10^{-5}\,\text {Pa}^{-1}$	greater cranial compliance

### Numerical methods & software

The complete system was solved via a fully coupled strategy with a an implicit Euler finite difference discretization in time and a finite element method in space, following [47]. We approximate the vector-valued unknowns, i.e. the tissue displacement and fluid velocity, with continuous piecewise quadratic polynomials, while continuous piecewise linear functions are employed for the pore pressure, total pressure, and fluid pressure. The model is implemented with the finite element software *FEniCS* [1] and its extension to multiphysics problems *multiphenics* [6]. The resulting linear system is factorized and solved in every time step with the direct solver *MUMPS* [3, 4], employing a hybrid approach of distributed and shared memory parallelism (via OpenMP and MPI).

We performed convergence tests against smooth manufactured solutions to verify the accuracy of the discretization and further verified the computations using mesh and time step convergence tests (Additional file 1: Figure S1 and Figure S2).

## Results

The validation of results by comparison with measurement data is crucial in mathematical modelling. For this reason, we first present the model output in terms of intracranial pressure, velocities and displacements in this section and continue with a detailed comparison of the results with clinical data and other modelling studies in the Discussion Section.

The cardiac-induced influx of blood to the brain parenchyma induces a complex interplay between the CSF-filled spaces and poroelastic parenchyma in terms of intracranial pressures and pressure gradients, CSF and ISF flow, and parenchymal displacements.

### Intracranial pressure

**Fig. 2:**
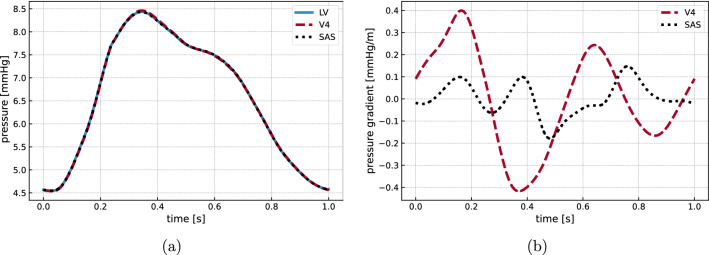
**a** Time evolution of the ICP at different locations: inside the lateral ventricles (LV), in the cranial SAS at the upper convexity of the skull (SAS), and inside the fourth ventricle (V4) (cf. Figure 1c). **b** Intracranial pressure gradient from the lateral ventricles to the upper convexity of the cranial SAS (black) and the fourth ventricle (red)

At the beginning of the cardiac cycle, the ICP rapidly and rises nearly uniformly in space from its initial value of 4.5 mmHg to reach a peak of 8.4 mmHg after approximately 0.3 s (Fig. 2). Subsequently, it steadily decreases until the initial value is reached again and the next cycle begins. The nadir-to-peak pressure variation in time is close to 4.0 mmHg, whereas the spatial differences are several orders of magnitude smaller. The transmantle pressure gradient between the lateral ventricles and upper convexity of the SAS peaks at 0.18 mmHg/m (Fig. 2b). The maximal gradient between the lateral and the fourth ventricle is almost three times larger, reaching a peak value of 0.41 mmHg/m. Within each cardiac cycle, the pressure gradient reverses multiple times.

**Fig. 3:**
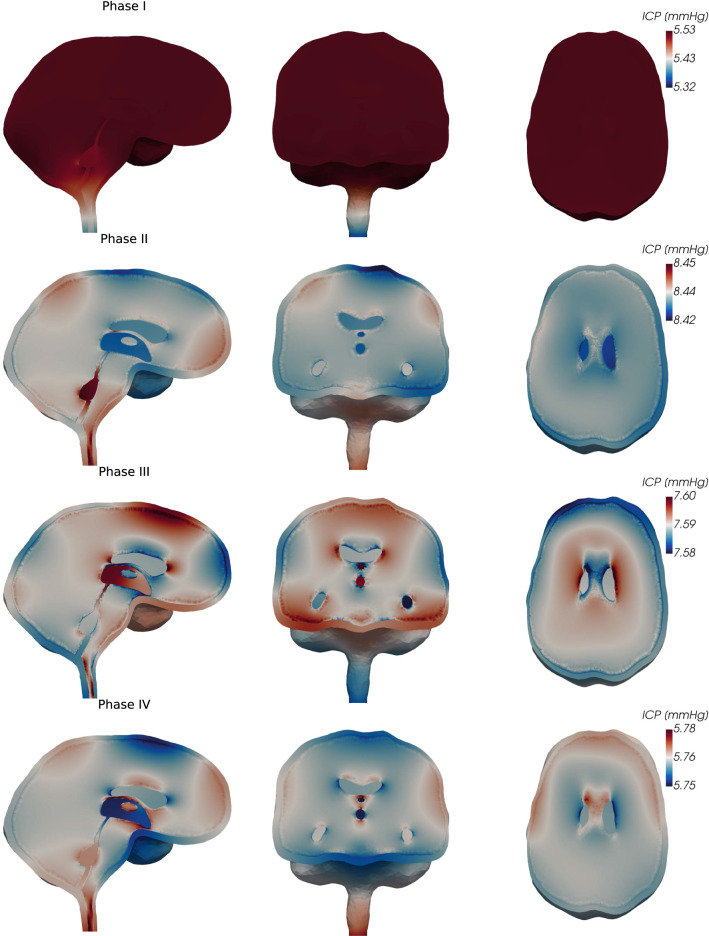
Sagittal, coronal, and transversal views of the ICP (fluid pressure in the CSF-filled spaces and total pressure in the parenchyma) during phases I–IV of the cardiac cycle. Note that the color scale changes between the different phases (rows)

The spatial ICP distribution differs between the four phases of the cardiac cycle (see Fig. 3 sagittal, coronal and transversal views). In phase I (early systole), we observe the largest spatial pressure variation of the four phases. While the ICP in the parenchyma, the ventricular system, and the cranial SAS are nearly equal (Fig. 2a), ICP decreases in the dorsal direction from the craniospinal junction at the foramen magnum. This results in a pressure drop of 0.21 mmHg from the cranium to the spinal compartment. Additionally, we observe a slightly lower pressure in the fourth ventricle compared to the third ventricle and surrounding tissue. In phase II (end of net blood inflow), spatial ICP differences amount to 0.03 mmHg, less than 15% of that of phase I. The peak pressure is now observed at the lowest point of the cervical spine and in the fourth ventricle. The pressure differences in the ventricular system thus reverse: highest values occur in the fourth ventricle, decreasing towards the third ventricle and resulting in a small pressure gradient over the aqueduct. Next, phase III (brain equilibrium) is characterized by small spatial pressure differences of less than 0.02 mmHg. Inside the ventricular system, the pressure difference over the aqueduct once again reverses, and the largest pressure is obtained in the third ventricle. Finally, in phase IV (high net blood outflow), the pressure increases from the craniocervical junction in the caudal direction. The lowest pressure occurs at the frontal part of the upper convexity of the skull and in the third ventricle. The pressure difference across the aqueduct reverses yet again.

### CSF flow patterns

**Fig. 4:**
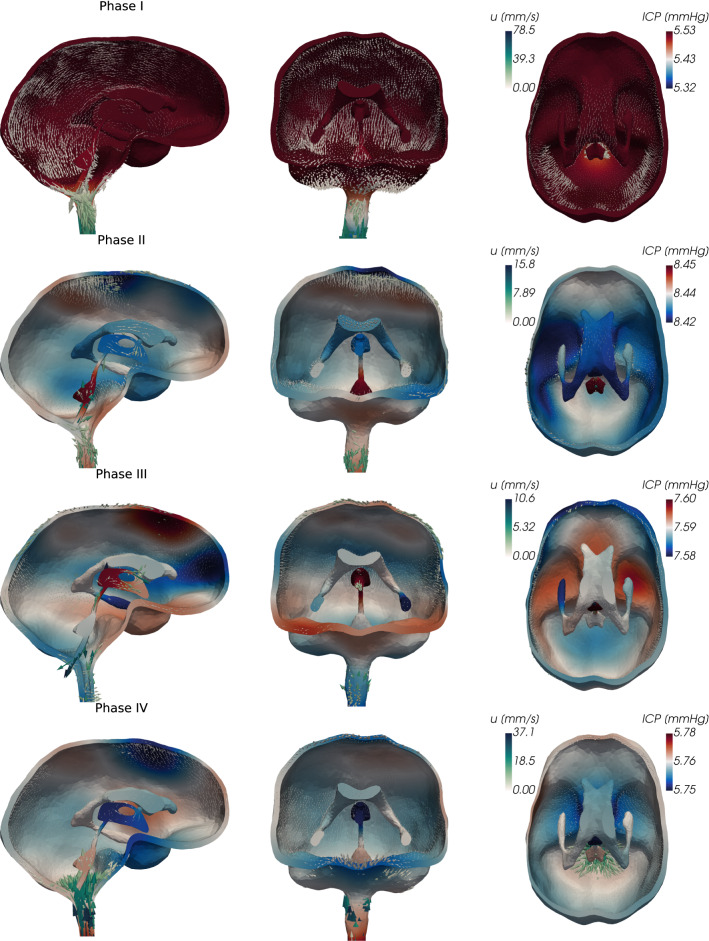
Sagittal, coronal, and tranverse views of the pressure (ICP) and fluid velocity ${\mathbf {u}}$ in the CSF-filled spaces of the cranium during different phases of the cardiac cycle. (Logarithmic scaling of the arrows representing the velocity)

The differences in pressure distributions induce characteristically different CSF flow patterns across the cardiac phases (Fig. 4). In phase I, CSF rushes out of the cranium into the spinal canal reaching a peak velocity of 78.5 mm/s at the craniocervical junction. Simultaneously, a slower, caudally-directed flow of CSF occurs within the cranial SAS at velocity magnitudes on the order of 10 mm/s. CSF inside the ventricular system is displaced downwards through the fourth ventricle and the median aperture. During phase 2, CSF flows from the lateral ventricles through the foramina of Monro into the third ventricle. Flow in the aqueduct is nearly stagnant, while flow in the median aperture reverses and is directed into the fourth ventricle. Simultaneously, the caudal CSF flow in the upper convexity of the cranium and the outflow into the spinal compartment continue on a smaller scale. In phase 3, almost no flow occurs into the spinal compartment. Inside the ventricular system, we again observe a reversal of flow directions: CSF moves in the median aperture in the dorsal direction and runs in the opposite direction at the level of the aqueduct and third ventricle. Finally, in phase 4, we observe the return of CSF from the spinal compartment into the cranium. CSF flows through the spinal canal, the cranial SAS, and the lower part of the ventricular system and thereby completes its cycle.

**Fig. 5:**
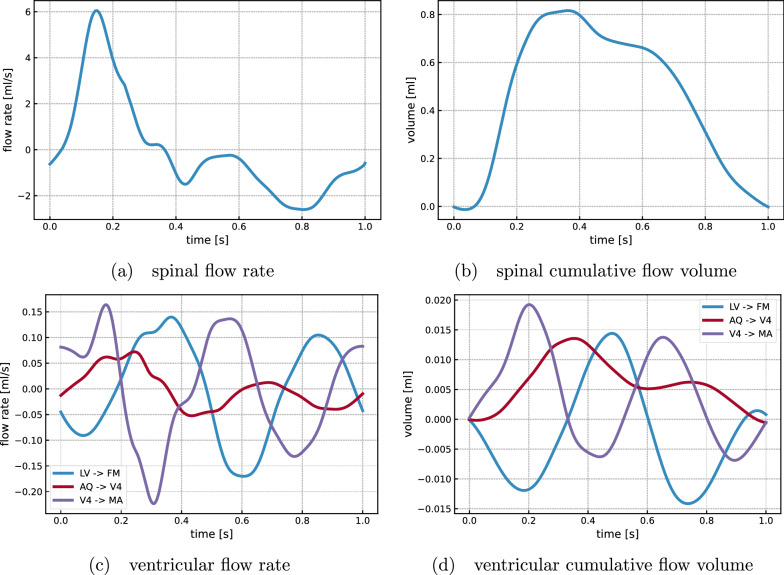
Volumetric flow rates and stroke volumes within the ventricular system and into the spinal compartment. LV -> FM denotes flow from the lateral ventricles into the foramina of Monro, AQ -> V4 from the aqueduct into the fourth ventricle, and V4 -> MA from the aqueduct into the median aperture (cf. Figure 1c)

In addition to this global description of CSF flow, we consider the flow rates and volumes in the ventricular system and at the cervical level in more detail (Fig. 5). Here, we employ flow rates and volumes instead of velocities, since fluid velocities are more sensitive to numerical and measurement errors as well as geometrical variations. However, average velocities can be computed using the diameters listed in Table 1. The largest flow rate occurs into the spinal canal, where up to 6 ml/s leave the cranium into the spinal compartment (Fig. 5a). This CSF-spinal flow rate thus corresponds to approximately two-thirds of the amplitude of the net blood inflow. The resulting stroke volume is 0.8 ml and corresponds to the peak value of the spinal cumulative flow volume at 35% of the cardiac cycle (Fig. 5b). The ventricular flow rates are at least one order of magnitude lower than those of the spinal canal, reaching at most 0.22 ml/s at the transition from the fourth ventricle to the median aperture. In the aqueduct, we observe a peak flow rate of 0.07 ml/s and a stroke volume of 0.013 ml (Fig. 5c, d). Notably, within each cardiac cycle, the flow reverses its direction multiple times. In the lower parts of the ventricular system (median aperture, aqueduct), flow initially takes place in the dorsal direction and changes its direction three times. At the level of the foramina of Monro, we observe a short phase of flow into the lateral ventricles at the beginning of the cycle and again three reversals of direction. Thus, the time of the flow rate peaks in the upper regions of the ventricular system are delayed compared to the lower regions (Fig. 5c).

Interstitial flow velocities and volumes within the parenchymal tissue pulsate with the cardiac cycle but are generally small (peak velocity magnitude less than 1.9 $\mu$m/s, and peak spatial average of 0.13 nm/s). The exchange between ISF and CSF is on the order of nanoliters per second which is negligible compared to flow rates in the spinal canal (on the order of ml/s).

### Brain parenchyma displacements

**Fig. 6:**
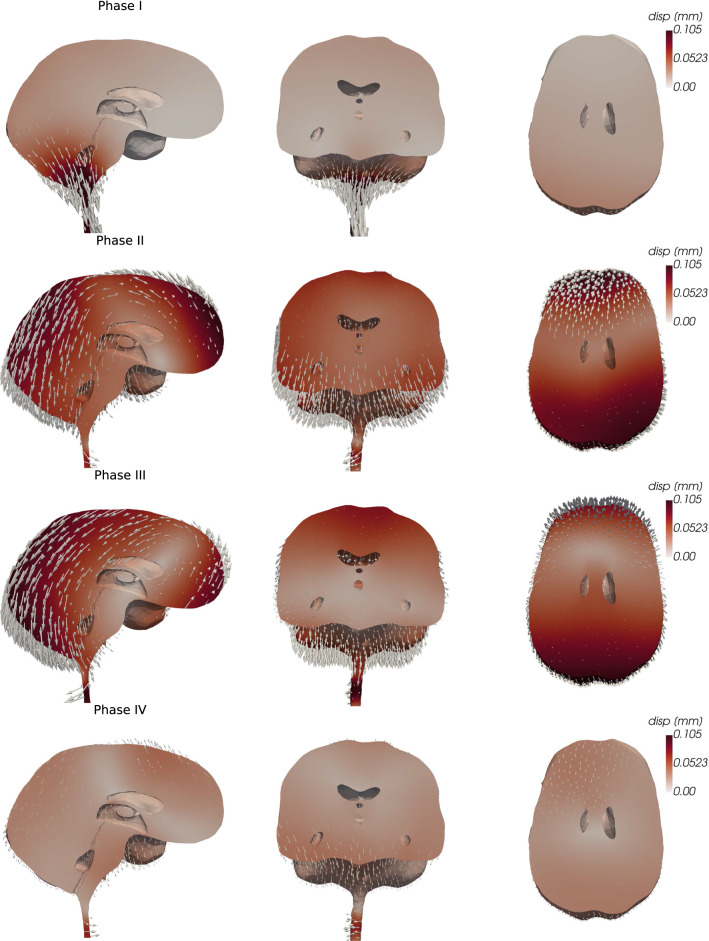
Sagittal, coronal, and transverse views of the brain parenchymal displacement during different phases of the cardiac cycle. The glyph arrows representing the displacement are amplified by a factor of 200

During early systole (phase I), a large dorsal deformation occurs, especially of the infratentorial part of the brain (Fig. 6). A peak displacement magnitude of 0.22 mm is found in the brain stem 12 % into the cardiac cycle. After $35\%$ of the cycle (in phase II), most of the infratentorial brain regions have return to their original configuration. In this phase, the displacement predominately occurs at the anterior and posterior ends, and we observe a rotational movement of the brain around its center. While the posterior regions are deformed downwards, the frontal region moves up and backwards. In the third phase, the overall pattern changes only slightly. Specifically, the anterior displacement decreases and the center of rotation moves forward. In the final phase of the cardiac cycle, the displacement magnitude decreases substantially and the remaining displacement is predominantly in the frontal superior parts in an upwards direction and in the central inferior region of the brain in the caudal direction. Throughout the cycle, we note some radial displacements of the spinal cord.

### Role of brain and spinal cord compliances

During the net inflow phase of blood, a total of 1.54 mL of fluid was added to the intracranial volume by the source *g*. While 0.83 mL of fluid was displaced into the spinal canal, the remaining 0.69 mL was stored withing the brain parenchyma due to the storage capacity (*c*). Thus 54% of the added fluid was accounted for by compliance along the spinal canal, while the remaining 46% was accounted for by intracranial compliance.

### Model variations

**Fig. 7:**
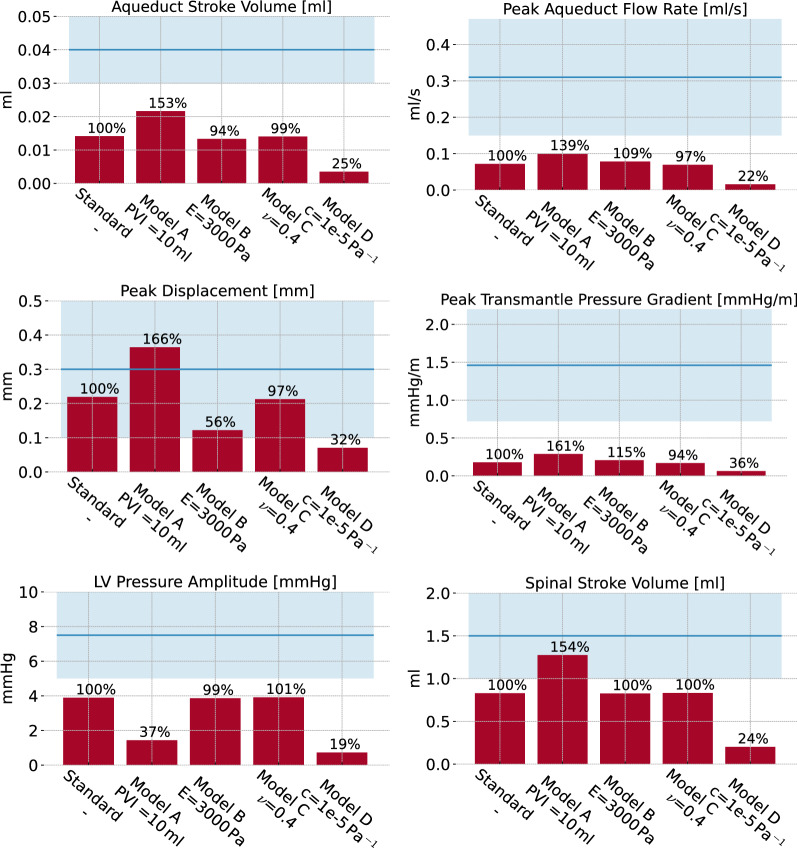
Overview of clinical quantities of interest of a set of model variations from the standard parameterization. The blue horizontal bar represents the range of physiologically realistic values with the blue line indicating the mean value

The set of quantities of interest predicted by the different computational models (models A–D) differ from the standard model (Fig. 7). For all quantities of interest, the outputs of the models range between 19 % and 166 % relative to the standard model.

Increased spinal compliance Increasing the spinal compliance by increasing the spinal pressure-volume index (Model A), yields increased aqueduct stroke volumes and peak aqueduct flow rates (by 53%, 39% respectively) relative to the standard model. The spinal stroke volumes increased by 54% (to 1.28 mL), and thus 83% of the total compliance was accounted for by the spinal canal. In addition, the peak displacement is increased by 66%, and the peak transmantle pressure gradient increases by 61%. Conversely, the total pressure variation in the lateral ventricles is substantially reduced, by 63%. In addition, the ICP curve changes characteristics (Additional file 1: Figure S3). With increased spinal compliance, additional peaks (P1, P2, P3) are seen in the ICP signal.

Increased brain stiffness Increasing the brain stiffness (Model B) reduces the peak brain displacement by 44% . The other clinical quantities of interest remain unchanged.

Increased brain compressibility Increasing the brain compressibility (Model C) yields only negligible changes in clinical quantities of interest. The largest difference relative to the standard model is observed for the peak transmantle pressure gradient, and only amounts to a 6% decrease.

Increased storage coefficient Decreasing the brain parenchyma’s poroelastic storage coefficient (Model D) results in substantial decreases in the set of clinical quantities of interest computed. The aqueduct stroke volume and peak aqueduct flow rates are reduced by by 75%, and 78%, respectively. The spinal stroke volumes decreased by 76% (to 0.20 mL), and thus only 13% of the total compliance was accounted for by the spinal canal. The peak displacement is decreased by 68%, and the peak transmantle pressure gradient by 64%. Similarly, the total pressure variation in the lateral ventricles is reduced by 81%.

## Discussion

We have presented a three-dimensional computational model of fully coupled cardiac-induced pulsatile CSF flow and tissue motion in the human brain environment. Variations in the ICP were dominated by their temporal amplitude, but with small spatial variations in both the CSF-filled spaces and the parenchyma. The ICP variations induce substantial ventricular and cranial-spinal CSF flow, some flow in the cranial SAS, and small pulsatile ISF velocities in the brain parenchyma. Investigating the displacement of parenchymal tissue, we found a funnel-shaped deformation in dorsal direction at the beginning of the cardiac cycle, followed by a rotational motion around an axis normal to the brain’s sagittal plane. Moderate variations in the brain and spinal cord compliances altered model outputs.

The temporal pressure variations are in good agreement with previous clinical reports. Wagshul et al [62] reported typical nadir-to-peak ICP amplitudes of 5 to 10 mmHg for healthy subjects, which is only slightly higher than the 4 mmHg obtained here. Considering the morphology of the ICP waveform, notable differences between individuals seem to exist: while the general cardiac cycle pattern of increasing and decreasing pressure persists across subjects, many clinical studies have reported several peaks in the ICP signal, often referred to as percussion wave (P1), tidal wave (P2) and dicrotic wave (P3) [58, 62]. Unnerbäck et al [58] suggested that the percussion wave is caused by the rapid rise of blood inflow, while the following peaks may be related to subsequent resonance phenomena. Carrera et al [13] related P1 to peak arterial inflow, while P2 and P3 were related to peak values in cerebral arterial blood volume. In our (standard) computational model, the peak in ICP signal is related to the change of sign in the net blood flow curve. However, additional peaks (P1, P2, P3) occur when the spinal compliance is increased. We note that our computed ICP curve lies well within the range of clinically reported curves by Ziółkowski et al [64] and closely resembles the in-vitro modelling results by Benninghaus et al [9]. A transmantle pressure gradient is hypothesized to drive the development of hydrocephalus [20, 52], though with recent findings also pointing at genetic factors [18]. Stephensen et al [52] reported no static transmantle pressure gradient, which agrees with the small pulsatile pressure gradients (peaking at $0.06-0.30$ mmHg/m) predicted here. Taking the pulsatile nature of the ICP into account, Eide [20] measured higher amplitudes in the lateral ventricles compared to the parenchymal tissue close to the skull. Similarly, Vinje et al [61] found pulsatile ICP gradients with average amplitudes of $1.46 \pm 0.74$ mmHg/m, which is roughly one order of magnitude higher than the pulsatile transmantle gradient obtained in this work. Complementary to these clinical findings in (suspected) hydrocephalic patients, Linninger et al [36] used computational fluid dynamics to compute maximal transmantle pressure differences of 10 Pa in healthy, and 30 Pa in hydrocephalus patients. In a subsequent modeling paper Sweetman et al [53] predicted a maximal transmantle pressure difference in healthy individuals of 4 Pa. Assuming a distance of 6 cm between the lateral ventricles and the SAS, these pressure differences correspond to pressure gradients of approximately 0.5–1.25 mmHg/m for healthy individuals and 3.75 mmHg/m for hydrocephalus patients. The computed transmantle pressure gradient is likely influenced by a number of model choices including the geometry representation, material parameters and importantly the assumed homogeneous net blood flow.

Consistent with the comparatively small spatial pressure differences computed, we find flow rates and stroke volumes in the ventricular system at the lower range of previous reports. The peak aqueductal flow rate and the spinal stroke volume of our standard model reach $70\,\%$ and $80\,\%$, respectively, of the values reported by Wagshul et al [62]. However, with a higher spinal compliance, the computed spinal stroke volume (1.25 ml) is within the clinical range. This finding represents a different distribution of compliance in the overall system: a higher spinal compliance allows more CSF to leave the cranium into the spinal compartment. In particular the contribution of the spinal canal to the overall cranio-spinal compliance was 54% in the standard model, but ranged between 13% and 83% (Model A and D). In line with our findings, values found in the literature range from 35% [63] to 69% [54] with large individual variability. Furthermore, our computed aqueduct stroke volume (13 $\mu$l) is lower than measured values of 30 to 50 $\mu$l [62]. Balédent [5] suggested that the contribution of the ventricular system to the regulation of ICP is low compared to the effect of cervical CSF outflow. This conforms with our results since the aqueductal flow peaks later than the cervical outflow and reaches only 16 % of its volume. The phase shift of ventricular CSF oscillations observed in the numerical results is in good agreement with clinical data. Balédent [5] found a significant phase shift between aqueductal and cervical CSF flow and Wagshul et al [62] reported a delay of 15% of the cardiac cycle in the cerebral aqueduct, which matches the 12% delay between peak aqueductal flow and peak blood inflow in our results. The peak spinal flow rate in our model reached 360 mL/min, while 150 mL/min has been reported in healthy subjects [2]. However, blood flow in our experimental data was also higher than in the study by Alperin et al. [2], and in both cases the peak spinal flow rate of CSF reached 2/3 of the peak net blood flow. The time course characteristics of spinal CSF flow rate compares well with reported time series in the same study [2]. Note that we emphasize a comparison between computational and clinical flow rates and volumes rather than CSF velocities, as velocities are more sensitive to numerical and measurement errors as well as unresolved geometrical features.

Balédent [5] observed the reversal of cervical CSF flow at the brain equilibrium phase at approximately $55\,\%$ of the cardiac cycle. In contrast, in our numerical results, the flow reverses after $38\,\%$ of the cycle, which corresponds to the begin of net blood outflow of the cranium. Additionally, their outflow curves take a smooth single-peaked shape over the cardiac cycle, while our results indicate a close resemblance of the flow rate curve and the blood inflow curve. This discrepancy may be explained by a lack of sufficient compliance in the modeled cranial system, which leads to a direct transfer of blood inflow to cervical CSF outflow morphology. Similarly, the multiple reversals of ventricular flow in our model do not match the clinically observed, almost sinusoidal waveforms [5]. These flow reversals are also expected to reduce the corresponding stroke volume. This behaviour might be explained by deviating elastic properties of the brain tissue, leading to multiple oscillations of pressure and flow after the initial excitation of the system at peak blood inflow.

Our model predicts peak ISF velocity magnitudes in agreement with reported values for interstitial bulk flow on the order of micrometers per second [40]. However, the ISF flow computed is pulsatile in time (representing back-and-forth motion over the cardiac cycle rather than bulk flow), and its spatial average is more than two orders of magnitude smaller than its peak value.

The magnitude and direction of the displacement are in good agreement with clinical findings. Based on MRI techniques, Enzmann and Pelc [22], Greitz et al [25] and Poncelet et al [44] reported the peak displacement of brain tissue to range from 0.1 to 0.5 mm. More recently, Pahlavian et al [42] found a peak mean displacement of the brain’s substructures of up to $0.187\pm 0.05$ mm and Sloots et al [50] reported peak displacements of around 0.2 mm; both fit well with the maximal value of 0.22 mm observed in our study. Both these studies [42, 50] found largest displacements at the brain stem, aligning well with observations from our model. Greitz et al [25] described a funnel-shaped movement in the dorsal direction and hypothesized that the relatively low pressure below the foramen magnum during early systole induces this motion, which aligns with our numerical results.

Although the model of intracranial pulsatility developed in this work is highly detailed in terms of geometry and biophysical mechanisms, several limitations remain. First, the complex interplay of arterial blood inflow, intracranial dynamics, and venous outflow is simplified into a spatially uniform fluid source in the parenchymal tissue. While the equivalence of the effect of additional fluid volume justifies this approximation, it may still be necessary to include heterogeneities in the source term to account for differences in blood perfusion in different regions. Differentiating between fluid networks (such as blood and CSF/ISF) with different viscosities and permeabilities may lead to further insights on the interaction of fluids within the brain. Furthermore, even though the time series of net blood flow used in this study (from Balédent [5]) is representative for healthy adults, individual differences in shape and amplitude of the cerebral blood inflow might have a substantial influence on flow and pressure patterns. Note that the effect of intracranial dynamics on arterial and venous blood flow is inherently captured in the measured blood inflow data, but not computed by the model in the sense of a two-way coupling. We therefore consider incorporating the interaction of the expanding vasculature with the surrounding parenchymal tissue as well as a spatially resolved representation of the brain’s vasculature a promising direction of future research. As gravitational forces only add a static pressure gradient countered by hydrostatic forces, we expect its influence to be minor and do not account for it in the present study. We also neglect CSF or ISF production effects, here without loss of relevance, as any net flow of CSF from its sites of production to absorption is two orders of magnitude smaller than the cardiac induced pulsatile motion [61].

Additional limitations include the uncertainty associated with material parameters, and the assumption of spatial homogeneity in brain tissue, as white and gray matter and subregions likely possess different elastic properties [12]. We expect the effect of moderate heterogeneity on the computational quantities of interest to be relatively small in light of our results with increased elastic stiffness (model B). Furthermore, the boundary conditions describing the transition to the spinal compartment are based on simplifying assumptions. Incorporating a flow resistance to the spinal outflow boundary condition and relaxing the no-displacement assumption of the spinal cord are likely to affect the computational predictions, especially in the brain stem and spinal compartment, and also the pulsatile flow patterns in the aqueduct. Despite the high degree of spatial detail of our model, some features of the intracranial anatomy remain unresolved. As an example, we hypothesize that the tentorium cerebelli would stabilize the brain tissue and block CSF flow, potentially leading to higher pressure differences between the infratentorial and supratentorial regions of the brain.

## Conclusion

In summary, we have presented a new computational model of intracranial fluid flow and tissue motion during the cardiac cycle that offers high resolution and detail in both space and time, and is well-aligned with clinical observations. The model offers a qualitative and quantitative platform for detailed investigation of coupled intracranial dynamics and interplay, both under physiological and pathophysiological conditions.

## Supplementary Information

**Additional file 1: Figure S1**. Quantities of interest computed for a sequence of uniformly refined meshes (coarse, mid, fine) with a fixed number of time steps (320 time steps per cardiac cycle).

## Data Availability

The code used to generate and analyze the datasets during the current study are openly available in repository [14].

## References

[1] Alnæ M, Blechta J, Hake J, Johansson A, Kehlet B, Logg A, Richardson C, Ring J, Rognes ME, Wells GN. The FEniCS project version 15. Arch Numer Softw 2015. 10.11588/ans.2015.100.20553

[2] Alperin N, Lee SH, Sivaramakrishnan A, Hushek SG (2005). Quantifying the effect of posture on intracranial physiology in humans by MRI flow studies. J Magn Reson Imaging.

[3] Amestoy P, Duff IS, Koster J, L’Excellent JY (2001). A fully asynchronous multifrontal solver using distributed dynamic scheduling. SIAM J Matrix Anal Appl.

[4] Amestoy P, Buttari A, L’Excellent JY, Mary T (2019). Performance and scalability of the block low-rank multifrontal factorization on multicore architectures. ACM Trans Math Softw.

[5] Balédent O. Imaging of the cerebrospinal fluid circulation. In: Rigamonti D, editor. Adult hydrocephalus, Cambridge University Press, Cambridge; 2014. p. 121–138, 10.1017/CBO9781139382816.013, https://www.cambridge.org/core/product/identifier/9781139382816%23c03177-12-1/type/book_part.

[6] Ballerin F. Multiphenics—mathLab innovating with mathematics; 2020. https://mathlab.sissa.it/multiphenics.

[7] Barber TW, Brockway JA, Higgins LS (1970). The density of tissues in and about the head. Acta Neurol Scand.

[8] Beavers GS, Joseph DD (1967). Boundary conditions at a naturally permeable wall. J Fluid Mech.

[9] Benninghaus A, Balédent O, Lokossou A, Castelar C, Leonhardt S, Radermacher K (2019). Enhanced in vitro model of the CSF dynamics. Fluids Barriers CNS.

[10] Biot MA (1941). General theory of three-dimensional consolidation. J Appl Phys.

[11] Bloomfield I, Johnston I, Bilston L (1998). Effects of proteins, blood cells and glucose on the viscosity of cerebrospinal fluid. Pediatr Neurosurg.

[12] Budday S, Nay R, de Rooij R, Steinmann P, Wyrobek T, Ovaert TC, Kuhl E (2015). Mechanical properties of gray and white matter brain tissue by indentation. J Mech Behav Biomed Mater.

[13] Carrera E, Kim DJ, Castellani G, Zweifel C, Czosnyka Z, Kasprowicz M, Smielewski P, Pickard JD, Czosnyka M (2010). What shapes pulse amplitude of intracranial pressure?. J Neurotrauma.

[14] Causemann M, Vinje V, Rognes ME (2022). Human intracranial pulsatility during the cardiac cycle: a computational modelling framework. bioRxiv.

[15] Chazen JL, Dyke JP, Holt RW, Horky L, Pauplis RA, Hesterman JY, Mozley DP, Verma A (2017). Automated segmentation of MR imaging to determine normative central nervous system cerebrospinal fluid volumes in healthy volunteers. Clin imaging.

[16] Chou D, Vardakis JC, Guo L, Tully BJ, Ventikos Y (2016). A fully dynamic multi-compartmental poroelastic system: application to aqueductal stenosis. J Biomech.

[17] Cosgrove KP, Mazure CM, Staley JK (2007). Evolving knowledge of sex differences in brain structure, function, and chemistry. Biol Psychiatr.

[18] Duy. Impaired neurogenesis alters brain biomechanics in a neuroprogenitor-based genetic subtype of congenital hydrocephalus. Nature X; 2022. 10.1038/s41593-022-01043-3 PMC9664907 35379995

[19] Ehrhardt M. An introduction to fluid-porous interface coupling. Prog Comput Phys 2010;10.

[20] Eide PK (2008). Demonstration of uneven distribution of intracranial pulsatility in hydrocephalus patients. J Neurosurg..

[21] Eide PK, Sæhle T (2010). Is ventriculomegaly in idiopathic normal pressure hydrocephalus associated with a transmantle gradient in pulsatile intracranial pressure?. Acta Neurochir.

[22] Enzmann DR, Pelc NJ (1992). Brain motion: measurement with phase-contrast MR imaging. Radiology.

[23] Fedorov A, Beichel R, Kalpathy-Cramer J, Finet J, Fillion-Robin JC, Pujol S, Bauer C, Jennings D, Fennessy F, Sonka M, Buatti J, Aylward S, Miller JV, Pieper S, Kikinis R (2012). 3D Slicer as an image computing platform for the Quantitative Imaging Network. Magn Reson Imaging.

[24] Gholampour S (2018). FSI simulation of CSF hydrodynamic changes in a large population of non-communicating hydrocephalus patients during treatment process with regard to their clinical symptoms. PLOS ONE.

[25] Greitz D, Wirestam R, Franck A, Nordell B, Thomsen C, Ståhlberg F (1992). Pulsatile brain movement and associated hydrodynamics studied by magnetic resonance phase imaging: the Monro-Kellie doctrine revisited. Neuroradiology.

[26] Guo L, Vardakis JC, Lassila T, Mitolo M, Ravikumar N, Chou D, Lange M, Sarrami-Foroushani A, Tully BJ, Taylor ZA, Varma S, Venneri A, Frangi AF, Ventikos Y (2018). Subject-specific multi-poroelastic model for exploring the risk factors associated with the early stages of Alzheimer’s disease. Interface Focus.

[27] Guo L, Li Z, Lyu J, Mei Y, Vardakis JC, Chen D, Han C, Lou X, Ventikos Y (2019). On the validation of a multiple-network poroelastic model using arterial spin labeling MRI data. Front Comput Neurosci.

[28] Haines D, Mihailoff G. An overview of the brainstem. In: Fundamental neuroscience for basic and clinical applications. Elsevier; 2018. p. 152–159. 10.1016/B978-0-323-39632-5.00010-4, https://linkinghub.elsevier.com/retrieve/pii/B9780323396325000104.

[29] Holter KE, Kehlet B, Devor A, Sejnowski TJ, Dale AM, Omholt SW, Ottersen OP, Nagelhus EA, Mardal KA, Pettersen KH (2017). Interstitial solute transport in 3D reconstructed neuropil occurs by diffusion rather than bulk flow. Proc Natl Acad Sci.

[30] Howden L, Giddings D, Power H, Aroussi A, Vloeberghs M, Garnett M, Walker D (2008). Three-dimensional cerebrospinal fluid flow within the human ventricular system. Comput Methods Biomech Biomed Eng.

[31] Jolesz FA, editor. Intraoperative imaging and image-guided therapy. New York: Springer; 2014.

[32] Kurtcuoglu V, Jain K, Martin BA. Modelling of cerebrospinal fluid flow by computational fluid dynamics. In: Biomechanics of the brain. Springer; 2019. p. 215–241.

[33] Lee JJ, Piersanti E, Mardal KA, Rognes ME (2019). A mixed finite element method for nearly incompressible multiple-network poroelasticity. SIAM J Sci Comput.

[34] Lindstrøm EK, Ringstad G, Mardal KA, Eide PK (2018). Cerebrospinal fluid volumetric net flow rate and direction in idiopathic normal pressure hydrocephalus. NeuroImage Clin.

[35] Linninger A, Tsakiris C, Zhu D, Xenos M, Roycewicz P, Danziger Z, Penn R (2005). Pulsatile cerebrospinal fluid dynamics in the human brain. IEEE Trans Biomed Eng.

[36] Linninger AA, Xenos M, Zhu DC, Somayaji MR, Kondapalli S, Penn RD (2007). Cerebrospinal fluid flow in the normal and hydrocephalic human brain. IEEE Trans Biomed Eng.

[37] Linninger AA, Tangen K, Hsu CY, Frim D (2016). Cerebrospinal fluid mechanics and its coupling to cerebrovascular dynamics. Ann Rev Fluid Mech.

[38] Marmarou A, Shulman K, LaMorgese J (1975). Compartmental analysis of compliance and outflow resistance of the cerebrospinal fluid system. J Neurosurg.

[39] Mikelic A, Jäger W (2000). On the interface boundary condition of Beavers, Joseph, and Saffman. SIAM J Appl Math.

[40] Nicholson C (2001). Diffusion and related transport mechanisms in brain tissue. Rep Prog Phys.

[41] Oyarzúa R, Ruiz-Baier R (2016). Locking-free finite element methods for poroelasticity. SIAM J Numer Anal.

[42] Pahlavian SH, Oshinski J, Zhong X, Loth F, Amini R (2018). Regional quantification of brain tissue strain using displacement-encoding with stimulated echoes magnetic resonance imaging. J Biomech Eng.

[43] Pardridge WM (2011). Drug transport in brain via the cerebrospinal fluid. Fluids Barriers CNS.

[44] Poncelet BP, Wedeen VJ, Weisskoff RM, Cohen MS (1992). Brain parenchyma motion: measurement with cine echo-planar MR imaging. Radiology.

[45] Rangel-Castillo L, Gopinath S, Robertson CS (2008). Management of intracranial hypertension. Neurol Clin.

[46] Ringstad G, Lindstrøm EK, Vatnehol SAS, Mardal KA, Emblem KE, Eide PK (2017). Non-invasive assessment of pulsatile intracranial pressure with phase-contrast magnetic resonance imaging. PloS one.

[47] Ruiz-Baier R, Taffetani M, Westermeyer HD, Yotov I (2022). The biot-stokes coupling using total pressure: formulation, analysis and application to interfacial flow in the eye. Comput Methods Appl Mech Eng.

[48] Saffman PG (1971). On the boundary condition at the surface of a porous medium. Stud Appl Math.

[49] Schubert JJ, Veronese M, Marchitelli L, Bodini B, Tonietto M, Stankoff B, Brooks DJ, Bertoldo A, Edison P, Turkheimer FE (2019). Dynamic 11c-PiB PET shows cerebrospinal fluid flow alterations in Alzheimer disease and multiple sclerosis. J Nucl Med.

[50] Sloots JJ, Biessels GJ, Zwanenburg JJ (2020). Cardiac and respiration-induced brain deformations in humans quantified with high-field MRI. Neuroimage.

[51] Smith JH, Humphrey JA (2007). Interstitial transport and transvascular fluid exchange during infusion into brain and tumor tissue. Microvasc Res..

[52] Stephensen H, Tisell M, Wikkelsö C (2002). There is no transmantle pressure gradient in communicating or noncommunicating hydrocephalus. Neurosurgery..

[53] Sweetman B, Xenos M, Zitella L, Linninger AA (2011). Three-dimensional computational prediction of cerebrospinal fluid flow in the human brain. Comput Biol Med.

[54] Tain RW, Bagci AM, Lam BL, Sklar EM, Ertl-Wagner B, Alperin N (2011). Determination of cranio-spinal canal compliance distribution by MRI: methodology and early application in idiopathic intracranial hypertension. J Magn Reson Imaging JMRI..

[55] Tangen KM, Hsu Y, Zhu DC, Linninger AA (2015). CNS wide simulation of flow resistance and drug transport due to spinal microanatomy. J Biomech.

[56] Tully B, Ventikos Y (2009). Coupling Poroelasticity and CFD for Cerebrospinal Fluid Hydrodynamics. IEEE Trans Biomed Eng.

[57] Ulbrich EJ, Schraner C, Boesch C, Hodler J, Busato A, Anderson SE, Eigenheer S, Zimmermann H, Sturzenegger M (2014). Normative MR cervical spinal canal dimensions. Radiology.

[58] Unnerbäck M, Ottesen JT, Reinstrup P (2018). ICP curve morphology and intracranial flow-volume changes: a simultaneous ICP and cine phase contrast MRI study in humans. Acta Neurochir.

[59] Valnes LM, Schreiner J. Surface volume meshing toolkit (SVMTK). https://github.com/SVMTK/SVMTK. 2020.

[60] Vardakis JC, Guo L, Peach TW, Lassila T, Mitolo M, Chou D, Taylor ZA, Varma S, Venneri A, Frangi AF, Ventikos Y (2019). Fluid-structure interaction for highly complex, statistically defined, biological media: Homogenisation and a 3D multi-compartmental poroelastic model for brain biomechanics. J Fluids Struct..

[61] Vinje V, Ringstad G, Lindstrøm EK, Valnes LM, Rognes ME, Eide PK, Mardal KA (2019). Respiratory influence on cerebrospinal fluid flow—a computational study based on long-term intracranial pressure measurements. Sci Rep..

[62] Wagshul ME, Eide PK, Madsen JR (2011). The pulsating brain: a review of experimental and clinical studies of intracranial pulsatility. Fluids Barriers CNS.

[63] Wåhlin A, Ambarki K, Birgander R, Alperin N, Malm J, Eklund A (2010). Assessment of craniospinal pressure-volume indices. AJNR Am J Neuroradiol.

[64] Ziółkowski A, Pudełko A, Kazimierska A, Czosnyka Z, Czosnyka M, Kasprowicz M (2021). Analysis of relative changes in pulse shapes of intracranial pressure and cerebral blood flow velocity. Physiol Meas.

