# Supplementary material for: Human intracranial pulsatility during the cardiac cycle: a computational modelling framework
Source: Fluids Barriers CNS. 2022 Nov 1;19:84. doi: 10.1186/s12987-022-00376-2 (PMC9623946; doi:10.1186/s12987-022-00376-2)
Supplement: Supplementary file 1 — Additional file 1: Figure S1. Quantities of interest computed for a sequence of uniformly refined meshes (coarse, mid, fine) with a fixed number of time steps (320 time steps per cardiac cycle). [file 12987_2022_376_MOESM1_ESM.pdf]

Human intracranial pulsatility during the cardiac cycle: a computational modelling framework - Supplementary Material

Mesh & time convergence

A highly detailed mesh is required to adequately resolve the intricate geometry of the human brain and its environment. To ensure a sufficiently fine mesh, we uniformly refined our mesh twice and hence obtained three meshes with increasing resolution (coarse, mid and fine) (Figure S1). Computing the set of quantities of interest on all meshes reveals that the temporal pressure variation in the lateral ventricles and the spinal stroke volume do not change with the mesh resolution, while the aqueductal stroke volume and the peak aqueduct flow rate increase from the coarse to the mid resolution meshes, but remain almost constant in the next refinement stage. The peak displacement and peak transmantle pressure gradient exhibit small decreases from the mid to fine resolution meshes, indicating that further mesh refinement may be desirable. However, given the small changes and limited computational resources, we consider the numerical error acceptable. Similarly, we conduct a time step refinement study on the fine resolution mesh, computing the quantities of interest using 80, 160 and 320 time steps per cardiac cycle. While the temporal pressure variations in the lateral ventricles and the spinal stroke volume again stay constant over time step refinement, the aqueduct stroke volume, the peak aqueduct flow rate, the peak displacement and the peak transmantle pressure gradient slightly increase with the number of time steps.

Intracranial Pressure with increased spinal compliance

The ICP pressure curve of Model A (increased spinal compliance) shows a smaller nadir-to-peak amplitude compared to the standard model, but features multiple peaks (P1,P2,P3) per cardiac cycle, which can tentatively be classified as percussion wave, tidal wave and dicrotic wave (Figure S4).

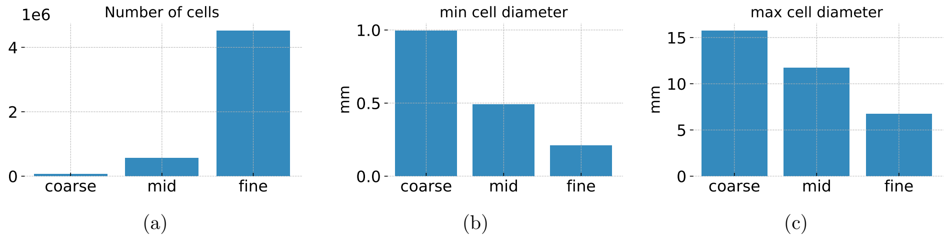

Figure S1: (a) number of cells of the fine, mid and coarse resolution meshes generated by uniform refinement); (b) minimal cell diameter of the meshes; (c) maximal cell diameter of the meshes

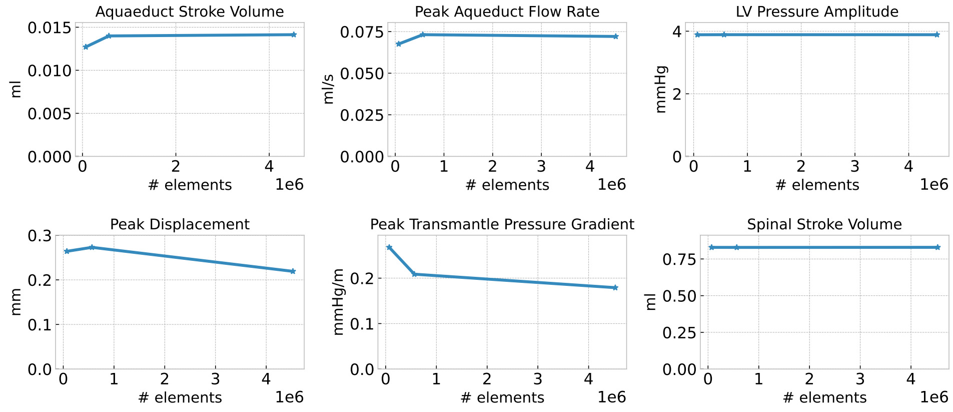

Figure S2: Quantities of interest computed for a sequence of uniformly refined meshes (coarse, mid, fine) with a fixed number of time steps (320 time steps per cardiac cycle).

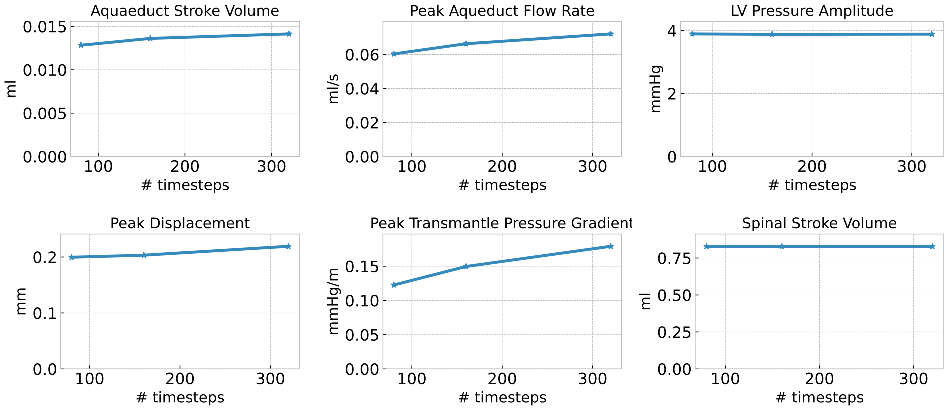

Figure S3: Quantities of interest computed for different numbers of time steps per cardiac cycle (80, 160, 320) on the fine mesh (uniformly refined twice).

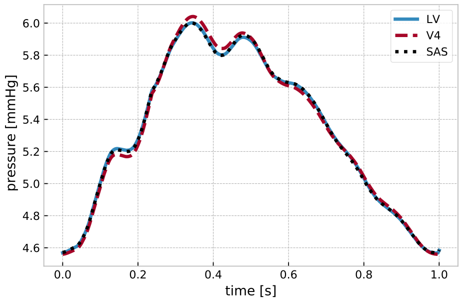

Figure S4: The ICP pressure curve in the lateral ventricles (LV) the fourth ventricle (V4) and the SAS of Model A (increased spinal compliance) shows multiple peaks per cardiac cycle (P1, P2, P3).
